# 1d-1-*O*-*tert*-Butyl­diphenyl­silyl-2,3,6-*O*-tris­(meth­oxy­methyl­ene)-*myo*-inositol 4,5-bis­(dibenzyl­phosphate)

**DOI:** 10.1107/S1600536812008069

**Published:** 2012-02-29

**Authors:** Regan J. Anderson, Graeme J. Gainsford

**Affiliations:** a Industrial Research Limited, PO Box 31-310, Lower Hutt, New Zealand

## Abstract

The title compound [systematic name: tetra­benzyl (1*R*,2*R*,3*S*,4*R*,5*R*,6*S*)-4-(*tert*-butyl­diphenyl­sil­yloxy)-3,5,6-tris­(meth­oxy­meth­oxy)cyclo­hexane-1,2-diyl bis­phosphate], C_56_H_68_O_15_P_2_Si, was isolated as an inter­mediate in the preparation of a phosphatidylinositol phosphate for biological studies. In the crystal, the mol­ecules are connected *via* one methyl­ene C—H⋯π and two weak phen­yl–ether C—H⋯O inter­actions. One benz­yloxy group is disordered over two overlapping positions with an occupancy ratio of 0.649 (7):0.351 (7).

## Related literature

For background material on the synthesis, see: Kubiak & Bruzik (2003 ▶). For structurally similar compounds, see: Bello *et al.* (2007 ▶); Sato *et al.* (2008 ▶). For the Cambridge Structural Database, see: Allen (2002 ▶). For hydrogen-bond motifs, see: Bernstein *et al.* (1995 ▶).
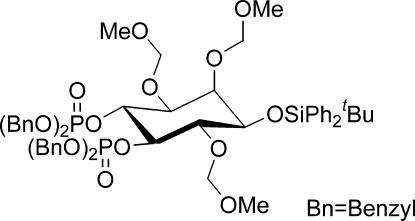


## Experimental

### 

#### Crystal data

C_56_H_68_O_15_P_2_Si
*M* *_r_* = 1071.13
Orthorhombic, 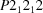

*a* = 10.4052 (7) Å
*b* = 53.019 (3) Å
*c* = 10.0786 (6) Å
*V* = 5560.1 (6) Å^3^
*Z* = 4
Mo *K*α radiation
μ = 0.17 mm^−1^
*T* = 118 K
0.50 × 0.42 × 0.05 mm

#### Data collection

Bruker APEXII CCD diffractometer
Absorption correction: multi-scan [*SADABS* (Bruker, 2005 ▶); Blessing (1995 ▶)] *T*_min_ = 0.600, *T*_max_ = 0.745
68347 measured reflections
8042 independent reflections
7297 reflections with *I* > 2σ(*I*)
*R*_int_ = 0.081

#### Refinement

*R*[*F* ^2^ > 2σ(*F* ^2^)] = 0.071
*wR*(*F* ^2^) = 0.181
*S* = 1.04
8042 reflections
655 parameters
16 restraints
H atoms treated by a mixture of independent and constrained refinement
Δρ_max_ = 0.38 e Å^−3^
Δρ_min_ = −0.43 e Å^−3^
Absolute structure: Flack (1983 ▶), 2324 Friedel pairs
Flack parameter: 0.12 (15)

### 

Data collection: *APEX2* (Bruker, 2005 ▶); cell refinement: *SAINT* (Bruker, 2005 ▶); data reduction: *SAINT* and *SADABS* (Bruker, 2005 ▶); program(s) used to solve structure: *SHELXS97* (Sheldrick, 2008 ▶); program(s) used to refine structure: *SHELXL97* (Sheldrick, 2008 ▶); molecular graphics: *ORTEP-3 for Windows* (Farrugia, 1997 ▶) and *Mercury* (Macrae *et al.*, 2008 ▶); software used to prepare material for publication: *SHELXL97* and *PLATON* (Spek, 2009 ▶).

## Supplementary Material

Crystal structure: contains datablock(s) global, I. DOI: 10.1107/S1600536812008069/gk2443sup1.cif

Structure factors: contains datablock(s) I. DOI: 10.1107/S1600536812008069/gk2443Isup2.hkl

Supplementary material file. DOI: 10.1107/S1600536812008069/gk2443Isup3.cml

Additional supplementary materials:  crystallographic information; 3D view; checkCIF report

## Figures and Tables

**Table 1:** Hydrogen-bond geometry (Å, °) *Cg*1 is the centroid of the C8–C13 ring.

*D*—H⋯*A*	*D*—H	H⋯*A*	*D*⋯*A*	*D*—H⋯*A*
C19—H19⋯O13^i^	0.95	2.50	3.342 (9)	148
C32*A*—H32*A*⋯O10^ii^	0.95	2.30	3.243 (11)	173
C14—H14*B*⋯*Cg*1^iii^	0.99	2.86	3.832 (7)	168

Symmetry codes: (i) 
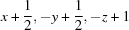
; (ii) 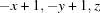; (iii) 
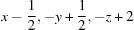
.

**Fig. 1.**
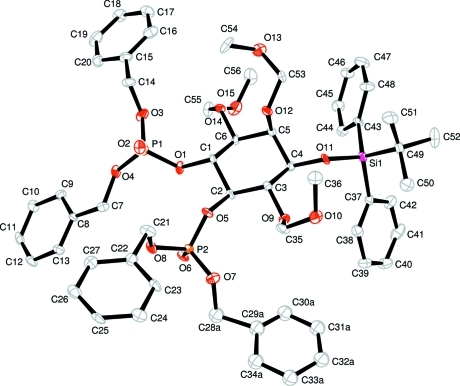
Asymmetric unit contents of the title compound; only the major conformer for benzyl atoms C28—C34 is shown (see text). H-atoms have been removed for clarity. Displacement ellipsoids are drawn at the 30% probability level.

**Fig. 2.**
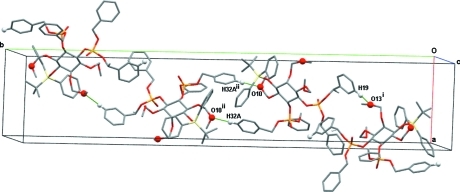
Packing diagram of (I) viewed down the c axis with hydrogen bonds shown as dashed lines (Macrae et al., 2008). H atoms not involved in intermolecular contacts (Table 1) are excluded. Symmetry operations: (i) 1/2 + x, 1/2 - y, 1 - z (ii) 1 - x, 1 - y, z.

## supplementary crystallographic information

### Comment

As part of a program to synthesize phosphatidylinositol phosphates for biological studies, the synthesis of phosphatidylinositol 4,5-bisphosphate was undertaken following a literature procedure (Kubiak & Bruzik, 2003). Crystals of an intermediate, the title compound (I), C_56_H_68_O_15_P_2_Si, were obtained from a hot EtOAc/petroleum ether (1:4) solution, after chromatographic purification.

The asymmetric unit of (I) contains one independent molecule of the title compound (Fig. 1). The absolute configuration of the molecule was indicated at low significance *by* anomalous dispersion effects and it confirmed the expected configuration.

One benzyloxy substituent (C28—C34) on atom P2 was disordered; only the major final model is shown in Figure 1. There is a wide variation in O–C (methylene) bond lengths (1.425 (7)–1.482 (7)) in the benzoyloxy chains but the average, and all other dimensions are consistent with previous reports of related compounds [CSD (Allen, 2002) codes TIXDUA (Sato *et al.*, 2008) and MIHYOS (Bello *et al.*, 2007)]. Crystal stabilization is provided by weak non-classical phenyl C–H···O and C–H···π interactions (Table 2, *Cg*1 is the centroid of ring C8–C13) building head to tail chains along the *b* axis. The key motifs (Bernstein *et al.*, 1995) are C(14) and *R*^2^_2_(28), the latter shown in Figure 2 involving the H32A···O10 interactions.

### Experimental

The title compound was prepared as described for compound 66 in Kubiak & Bruzik (2003). Crystals were obtained from a hot EtOAc/petroleum ether (1:4) solution after purification and isolation (mp: 368–369K). [α]^20^_D_ = +7.3 (c 2.0 g 100 m*L*^-1^, CHCl_3_); ^31^P NMR (202 MHz, CDCl_3_) δ -1.4, -1.1; HRMS(ESI) calcd for C_56_H_68_NaO_15_P_2_Si [*M*+Na] 1093.3700, found 1093.3712

### Refinement

Refinement of the final model gave conventional *R* (R1) of 12% with many data having *F_o_* >> Fc. This was consistent with overlap of data given the (unexpectedly large) length of the *c* axis, and the initial difficulty in defining the unit cell for data processing. It was not possible to recollect data using more suitable diffractometer settings or radiation wavelength. Data with I(obs) > *x**I(calc) and with I(obs)-I(calc) > *x**Sigma(Iobs) were removed from the dataset, progessively from *x* 2.0 to *x*=1.3. Using the remaining 8053 data from an *x* value of 1.45 gave a "worst agreement" table which indicated that most of the overlapped data had been removed: the 1977 reflections removed gave an R1 of 0.36. In refinement, 10 further reflections measured at low theta angle with I(obs) << I(calc) were removed as outliers.

The phenyl ring atoms C29—C34 (Figure 1) were disordered over two orientations dictated by the two-site disorder of C28. The two corresponding phenyl ring atom sets were located and refined with a total occupancy of 1.0 with each having a group and individual C–C distance constraint (AFIX 6 & DFIX) of 1.39 Å. All carbon atoms were given a common isotropic *U*_isod_ value and hydrogen atoms were added at expected positions with fixed U values of 1.5**U*_isod_. The C–H distances for the two C28 sites were refined with a C—H restraint of 0.99 (3) Å. Final group occupancies were 0.649 (7):0.351 (7) and the common carbon U was 0.0510 (11) Å^2^. All other carbon-bound H atoms were constrained to their expected geometries [C—H 0.95,0.98, 0.99 Å]. All methyl H atoms were free to rotate (HFIX 137). All methyl & disordered H/other H atoms were refined with *U*_iso_ 1.5/1.2 times the *U*_eq_ of their parent atom. All other non-hydrogen atoms were refined with anisotropic thermal parameters.

### Crystal data

**Table:**

C_56_H_68_O_15_P_2_Si	*F*(000) = 2272
*M**_r_* = 1071.13	*D*_x_ = 1.280 Mg m^−^^3^
Orthorhombic, *P*2_1_2_1_2	Mo *K*α radiation, λ = 0.71073 Å
Hall symbol: P 2 2ab	Cell parameters from 9935 reflections
*a* = 10.4052 (7) Å	θ = 2.3–24.9°
*b* = 53.019 (3) Å	µ = 0.17 mm^−^^1^
*c* = 10.0786 (6) Å	*T* = 118 K
*V* = 5560.1 (6) Å^3^	Triangular, colourless
*Z* = 4	0.50 × 0.42 × 0.05 mm

### Data collection

**Table:**

Bruker APEXII CCD diffractometer	8042 independent reflections
Radiation source: fine-focus sealed tube	7297 reflections with *I* > 2σ(*I*)
Graphite monochromator	*R*_int_ = 0.081
Detector resolution: 8.333 pixels mm^-1^	θ_max_ = 25.3°, θ_min_ = 2.5°
φ and ω scans	*h* = −12→12
Absorption correction: multi-scan [*SADABS* (Bruker, 2005); Blessing (1995)]	*k* = −63→62
*T*_min_ = 0.600, *T*_max_ = 0.745	*l* = −11→12
68347 measured reflections	

### Refinement

**Table:**

Refinement on *F*^2^	Secondary atom site location: difference Fourier map
Least-squares matrix: full	Hydrogen site location: inferred from neighbouring sites
*R*[*F*^2^ > 2σ(*F*^2^)] = 0.071	H atoms treated by a mixture of independent and constrained refinement
*wR*(*F*^2^) = 0.181	*w* = 1/[σ^2^(*F*_o_^2^) + (0.1107*P*)^2^ + 10.0351*P*] where *P* = (*F*_o_^2^ + 2*F*_c_^2^)/3
*S* = 1.04	(Δ/σ)_max_ = 0.007
8042 reflections	Δρ_max_ = 0.38 e Å^−^^3^
655 parameters	Δρ_min_ = −0.43 e Å^−^^3^
16 restraints	Absolute structure: Flack (1983), 2324 Friedel pairs
Primary atom site location: structure-invariant direct methods	Flack parameter: 0.12 (15)

### Special details

**Table:**

Experimental. 1H NMR (500?MHz, CDCl3) δ 1.11 (s, 9H), 2.96 (s, 3H), 3.19 (dd, J = 2.1, 10.0?Hz, 1H), 3.25 (s, 3H), 3.28 (t, J = 2.1?Hz, 1H), 3.38 (s, 3H), 3.86 (dd, J = 2.0, 9.7?Hz, 1H), 4.04 (d, J = 7.0?Hz, 1H), 4.19 (t, J = 9.6?Hz, 1H), 4.27–4.34 (m, 2H), 4.52 (d, J = 6.4?Hz, 1H), 4.58 (d, J = 6.4?Hz, 1H), 4.75–4.82 (m, 2H), 4.92 (dd, J = 6.5, 11.8?Hz, 1H), 4.97–5.11 (m, 7H), 5.14 (dd, J = 7.4, 11.9?Hz, 1H), 7.20–7.31 (m, 20H), 7.36–7.45 (m, 6H), 7.69–7.70 (m, 2H), 7.76–7.78 (m, 2H); 13C NMR (126?MHz, CDCl3) δ 19.2, 27.3, 55.6, 55.7, 57.0, 69.1 (d, J = 4.4?Hz), 69.3 (d, J = 4.4?Hz), 69.4 (d, J = 5.4?Hz), 69.5 (d, J = 5.2?Hz), 73.8, 74.2, 75.6, 75.9, 77.9, 78.6, 96.0, 97.4, 98.8, 127.80, 127.84, 127.9, 128.0, 128.1, 128.2, 128.3, 128.4, 130.0, 130.1, 132.6, 134.0, 135.9, 136.1, 136.26, 136.30, 136.4;
Geometry. All esds (except the esd in the dihedral angle between two l.s. planes) are estimated using the full covariance matrix. The cell esds are taken into account individually in the estimation of esds in distances, angles and torsion angles; correlations between esds in cell parameters are only used when they are defined by crystal symmetry. An approximate (isotropic) treatment of cell esds is used for estimating esds involving l.s. planes.
Refinement. Refinement of *F*^2^ against ALL reflections. The weighted *R*-factor *wR* and goodness of fit *S* are based on *F*^2^, conventional *R*-factors *R* are based on *F*, with *F* set to zero for negative *F*^2^. The threshold expression of *F*^2^ > σ(*F*^2^) is used only for calculating *R*-factors(gt) *etc*. and is not relevant to the choice of reflections for refinement. *R*-factors based on *F*^2^ are statistically about twice as large as those based on *F*, and *R*- factors based on ALL data will be even larger.

### Fractional atomic coordinates and isotropic or equivalent isotropic displacement parameters (Å^2^)

**Table:**

	*x*	*y*	*z*	*U*_iso_*/*U*_eq_	Occ. (<1)
P1	0.51021 (15)	0.30563 (3)	0.81745 (14)	0.0248 (3)	
P2	0.68940 (14)	0.37535 (3)	0.89914 (14)	0.0218 (3)	
Si1	0.22969 (15)	0.42782 (3)	0.40560 (16)	0.0236 (3)	
O1	0.5361 (4)	0.32847 (7)	0.7182 (4)	0.0238 (8)	
O2	0.4477 (4)	0.31308 (7)	0.9399 (4)	0.0300 (9)	
O3	0.4419 (5)	0.28550 (7)	0.7295 (4)	0.0340 (10)	
O4	0.6450 (4)	0.29329 (7)	0.8285 (4)	0.0263 (9)	
O5	0.5440 (3)	0.37372 (7)	0.8640 (3)	0.0220 (8)	
O6	0.7822 (3)	0.36617 (7)	0.8036 (4)	0.0270 (8)	
O7	0.7058 (4)	0.40374 (8)	0.9378 (4)	0.0362 (10)	
O8	0.6963 (4)	0.36004 (7)	1.0325 (4)	0.0271 (9)	
O9	0.4538 (4)	0.41785 (7)	0.7376 (4)	0.0237 (8)	
O10	0.3178 (4)	0.44486 (7)	0.8584 (5)	0.0381 (11)	
O11	0.2570 (4)	0.41287 (7)	0.5474 (4)	0.0227 (8)	
O12	0.1881 (4)	0.36476 (7)	0.6405 (4)	0.0262 (8)	
O13	0.0361 (4)	0.33465 (8)	0.5734 (4)	0.0357 (10)	
O14	0.3313 (4)	0.32276 (7)	0.5353 (4)	0.0226 (8)	
O15	0.3933 (6)	0.31597 (8)	0.3167 (5)	0.0481 (13)	
C1	0.4384 (5)	0.34754 (9)	0.7015 (5)	0.0206 (11)	
H1	0.3666	0.3447	0.7657	0.025*	
C2	0.5002 (5)	0.37315 (10)	0.7267 (5)	0.0197 (10)	
H2	0.5737	0.3760	0.6646	0.024*	
C3	0.3976 (5)	0.39422 (9)	0.7101 (5)	0.0203 (11)	
H3	0.3243	0.3911	0.7722	0.024*	
C4	0.3498 (5)	0.39319 (10)	0.5663 (5)	0.0220 (11)	
H4	0.4234	0.3954	0.5035	0.026*	
C5	0.2870 (5)	0.36739 (10)	0.5453 (5)	0.0209 (11)	
H5	0.2495	0.3665	0.4541	0.025*	
C6	0.3886 (5)	0.34639 (9)	0.5621 (6)	0.0223 (11)	
H6	0.4610	0.3493	0.4987	0.027*	
C7	0.7482 (6)	0.30298 (11)	0.9161 (7)	0.0331 (13)	
H7A	0.7143	0.3054	1.0070	0.040*	
H7B	0.7788	0.3195	0.8828	0.040*	
C8	0.8564 (5)	0.28471 (10)	0.9188 (5)	0.0215 (11)	
C9	0.8509 (6)	0.26185 (10)	0.8541 (6)	0.0288 (13)	
H9	0.7764	0.2573	0.8053	0.035*	
C10	0.9577 (6)	0.24519 (11)	0.8609 (6)	0.0318 (14)	
H10	0.9551	0.2294	0.8168	0.038*	
C11	1.0667 (7)	0.25229 (14)	0.9332 (6)	0.0375 (15)	
H11	1.1381	0.2412	0.9381	0.045*	
C12	1.0715 (6)	0.27491 (14)	0.9963 (7)	0.0364 (15)	
H12	1.1467	0.2798	1.0432	0.044*	
C13	0.9633 (6)	0.29117 (11)	0.9915 (6)	0.0292 (13)	
H13	0.9647	0.3067	1.0388	0.035*	
C14	0.3399 (6)	0.27005 (12)	0.7774 (6)	0.0347 (14)	
H14A	0.2628	0.2805	0.7936	0.042*	
H14B	0.3654	0.2622	0.8624	0.042*	
C15	0.3096 (7)	0.25028 (12)	0.6794 (6)	0.0352 (14)	
C16	0.2071 (7)	0.25236 (12)	0.5926 (7)	0.0437 (16)	
H16	0.1503	0.2663	0.5990	0.052*	
C17	0.1871 (7)	0.23391 (14)	0.4957 (7)	0.0407 (16)	
H17	0.1186	0.2357	0.4340	0.049*	
C18	0.2662 (8)	0.21320 (13)	0.4893 (6)	0.0450 (18)	
H18	0.2510	0.2006	0.4242	0.054*	
C19	0.3641 (9)	0.21062 (13)	0.5739 (7)	0.050 (2)	
H19	0.4165	0.1960	0.5692	0.060*	
C20	0.3909 (7)	0.22900 (12)	0.6690 (7)	0.0392 (15)	
H20	0.4630	0.2272	0.7261	0.047*	
C21	0.5946 (6)	0.36118 (14)	1.1330 (6)	0.0356 (15)	
H21A	0.5268	0.3730	1.1038	0.043*	
H21B	0.5552	0.3443	1.1434	0.043*	
C22	0.6467 (6)	0.36949 (11)	1.2602 (6)	0.0329 (14)*	
C23	0.6542 (6)	0.39494 (12)	1.2917 (6)	0.0306 (13)	
H23	0.6206	0.4071	1.2316	0.037*	
C24	0.7104 (6)	0.40299 (13)	1.4099 (7)	0.0382 (14)	
H24	0.7149	0.4204	1.4309	0.046*	
C25	0.7593 (6)	0.38516 (13)	1.4959 (6)	0.0340 (14)	
H25	0.7990	0.3905	1.5760	0.041*	
C26	0.7520 (6)	0.35983 (13)	1.4681 (7)	0.0368 (15)	
H26	0.7836	0.3479	1.5304	0.044*	
C27	0.6984 (6)	0.35154 (12)	1.3492 (6)	0.0329 (14)	
H27	0.6966	0.3341	1.3282	0.039*	
C35	0.4252 (6)	0.42804 (12)	0.8605 (7)	0.0353 (15)	
H35A	0.5014	0.4373	0.8938	0.042*	
H35B	0.4069	0.4141	0.9233	0.042*	
C36	0.1951 (7)	0.43296 (13)	0.8674 (7)	0.0409 (16)	
H36A	0.1874	0.4202	0.7976	0.061*	
H36B	0.1274	0.4456	0.8563	0.061*	
H36C	0.1862	0.4249	0.9544	0.061*	
C37	0.3809 (6)	0.44399 (10)	0.3504 (6)	0.0283 (13)	
C38	0.4702 (6)	0.45191 (12)	0.4419 (6)	0.0343 (14)	
H38	0.4585	0.4478	0.5328	0.041*	
C39	0.5775 (7)	0.46586 (13)	0.4043 (8)	0.0456 (17)	
H39	0.6381	0.4710	0.4693	0.055*	
C40	0.5963 (7)	0.47231 (14)	0.2713 (7)	0.0449 (17)	
H40	0.6705	0.4815	0.2448	0.054*	
C41	0.5031 (9)	0.46498 (12)	0.1769 (8)	0.054 (2)	
H41	0.5122	0.4696	0.0863	0.064*	
C42	0.4002 (8)	0.45125 (12)	0.2175 (6)	0.0393 (16)	
H42	0.3384	0.4463	0.1533	0.047*	
C43	0.1779 (6)	0.40495 (11)	0.2738 (5)	0.0254 (12)	
C44	0.2746 (6)	0.39225 (11)	0.1977 (6)	0.0305 (13)	
H44	0.3629	0.3959	0.2118	0.037*	
C45	0.2382 (7)	0.37443 (12)	0.1024 (7)	0.0429 (16)	
H45	0.3024	0.3664	0.0502	0.052*	
C46	0.1120 (6)	0.36830 (13)	0.0831 (7)	0.0369 (14)	
H46	0.0895	0.3561	0.0179	0.044*	
C47	0.0168 (7)	0.37972 (14)	0.1580 (8)	0.0475 (18)	
H47	−0.0709	0.3753	0.1454	0.057*	
C48	0.0506 (6)	0.39768 (12)	0.2512 (6)	0.0346 (14)	
H48	−0.0155	0.4054	0.3020	0.042*	
C49	0.1022 (7)	0.45170 (11)	0.4550 (7)	0.0361 (15)	
C50	0.1681 (7)	0.47292 (12)	0.5370 (7)	0.0394 (15)	
H50A	0.1029	0.4848	0.5690	0.059*	
H50B	0.2299	0.4819	0.4808	0.059*	
H50C	0.2131	0.4655	0.6129	0.059*	
C51	−0.0050 (7)	0.44043 (13)	0.5350 (8)	0.0434 (17)	
H51A	−0.0694	0.4534	0.5541	0.065*	
H51B	0.0294	0.4338	0.6186	0.065*	
H51C	−0.0450	0.4267	0.4847	0.065*	
C52	0.0453 (9)	0.46379 (14)	0.3285 (8)	0.058 (2)	
H52A	0.1152	0.4697	0.2714	0.086*	
H52B	−0.0095	0.4781	0.3533	0.086*	
H52C	−0.0060	0.4512	0.2806	0.086*	
C53	0.0641 (6)	0.36038 (11)	0.5964 (7)	0.0362 (15)	
H53A	0.0504	0.3699	0.5131	0.043*	
H53B	0.0030	0.3670	0.6631	0.043*	
C54	0.0393 (9)	0.32090 (13)	0.6974 (8)	0.055 (2)	
H54A	0.1287	0.3179	0.7232	0.083*	
H54B	−0.0051	0.3047	0.6865	0.083*	
H54C	−0.0037	0.3308	0.7665	0.083*	
C55	0.4009 (7)	0.30731 (12)	0.4478 (6)	0.0356 (15)	
H55A	0.4920	0.3069	0.4758	0.043*	
H55B	0.3668	0.2899	0.4525	0.043*	
C56	0.2721 (8)	0.31194 (13)	0.2592 (7)	0.060 (2)	
H56A	0.2408	0.2951	0.2835	0.089*	
H56B	0.2792	0.3131	0.1624	0.089*	
H56C	0.2117	0.3247	0.2914	0.089*	
C28A	0.8291 (8)	0.41653 (17)	0.9201 (10)	0.0511 (10)*	0.649 (7)
H28A	0.8995	0.4050	0.8941	0.077*	0.649 (7)
H28B	0.8310	0.4188	1.0176	0.077*	0.649 (7)
C30A	0.7303 (12)	0.4523 (2)	0.7929 (11)	0.0511 (10)*	0.649 (7)
H30A	0.7042	0.4409	0.7255	0.077*	0.649 (7)
C31A	0.7039 (12)	0.47780 (19)	0.7838 (12)	0.0511 (10)*	0.649 (7)
H31A	0.6531	0.4835	0.7115	0.077*	0.649 (7)
C32A	0.7474 (12)	0.4954 (2)	0.8739 (11)	0.0511 (10)*	0.649 (7)
H32A	0.7292	0.5128	0.8617	0.077*	0.649 (7)
C33A	0.8183 (13)	0.48738 (19)	0.9826 (12)	0.0511 (10)*	0.649 (7)
H33A	0.8490	0.4990	1.0471	0.077*	0.649 (7)
C34A	0.8425 (11)	0.46165 (17)	0.9935 (10)	0.0511 (10)*	0.649 (7)
H34A	0.8933	0.4559	1.0658	0.077*	0.649 (7)
C29A	0.7966 (11)	0.44406 (17)	0.9048 (10)	0.0511 (10)*	0.649 (7)
C28B	0.773 (2)	0.4201 (4)	0.834 (2)	0.0511 (10)*	0.351 (7)
H28C	0.862 (10)	0.413 (4)	0.83 (3)	0.077*	0.351 (7)
H28D	0.701 (17)	0.418 (5)	0.77 (2)	0.077*	0.351 (7)
C30B	0.715 (2)	0.4629 (3)	0.811 (2)	0.0511 (10)*	0.351 (7)
H30B	0.6721	0.4568	0.7345	0.077*	0.351 (7)
C31B	0.700 (2)	0.4885 (3)	0.843 (2)	0.0511 (10)*	0.351 (7)
H31B	0.6461	0.5000	0.7964	0.077*	0.351 (7)
C32B	0.776 (2)	0.4946 (4)	0.952 (2)	0.0511 (10)*	0.351 (7)
H32B	0.7751	0.5119	0.9761	0.077*	0.351 (7)
C33B	0.851 (2)	0.4793 (3)	1.032 (2)	0.0511 (10)*	0.351 (7)
H33B	0.8922	0.4853	1.1097	0.077*	0.351 (7)
C34B	0.864 (2)	0.4544 (3)	0.989 (2)	0.0511 (10)*	0.351 (7)
H34B	0.9230	0.4434	1.0307	0.077*	0.351 (7)
C29B	0.788 (3)	0.4460 (4)	0.884 (2)	0.0511 (10)*	0.351 (7)

### Atomic displacement parameters (Å^2^)

**Table:**

	*U*^11^	*U*^22^	*U*^33^	*U*^12^	*U*^13^	*U*^23^
P1	0.0329 (8)	0.0219 (6)	0.0197 (7)	0.0017 (6)	−0.0025 (6)	0.0005 (6)
P2	0.0250 (7)	0.0244 (7)	0.0161 (6)	−0.0008 (5)	−0.0038 (6)	0.0005 (5)
Si1	0.0236 (7)	0.0250 (7)	0.0223 (7)	0.0031 (6)	−0.0054 (6)	0.0032 (6)
O1	0.029 (2)	0.0209 (18)	0.0217 (19)	0.0122 (16)	0.0082 (17)	0.0038 (15)
O2	0.041 (2)	0.031 (2)	0.0181 (19)	0.0073 (18)	0.0021 (17)	−0.0038 (16)
O3	0.052 (3)	0.0198 (19)	0.030 (2)	−0.0074 (18)	0.000 (2)	−0.0065 (17)
O4	0.026 (2)	0.0252 (19)	0.028 (2)	0.0110 (15)	0.0055 (17)	0.0037 (16)
O5	0.0150 (18)	0.033 (2)	0.0177 (18)	0.0020 (16)	−0.0040 (14)	−0.0053 (15)
O6	0.0120 (18)	0.039 (2)	0.030 (2)	0.0025 (15)	−0.0044 (16)	−0.0010 (17)
O7	0.038 (3)	0.030 (2)	0.040 (3)	−0.0069 (19)	−0.001 (2)	−0.0041 (18)
O8	0.0147 (19)	0.037 (2)	0.030 (2)	0.0088 (16)	0.0029 (17)	0.0048 (17)
O9	0.027 (2)	0.0217 (18)	0.0223 (19)	−0.0024 (15)	0.0008 (16)	−0.0016 (15)
O10	0.032 (2)	0.025 (2)	0.057 (3)	0.0063 (18)	0.008 (2)	−0.0066 (19)
O11	0.0223 (19)	0.027 (2)	0.0189 (18)	0.0041 (16)	−0.0114 (16)	0.0016 (15)
O12	0.0164 (18)	0.033 (2)	0.029 (2)	0.0001 (16)	−0.0016 (16)	0.0069 (16)
O13	0.032 (2)	0.037 (2)	0.038 (3)	0.0005 (19)	0.012 (2)	−0.0053 (19)
O14	0.021 (2)	0.0236 (18)	0.0230 (19)	−0.0028 (15)	−0.0038 (16)	−0.0062 (15)
O15	0.086 (4)	0.032 (2)	0.026 (2)	−0.013 (2)	−0.012 (3)	−0.0082 (19)
C1	0.023 (3)	0.021 (3)	0.017 (3)	0.003 (2)	−0.005 (2)	0.004 (2)
C2	0.014 (2)	0.031 (3)	0.014 (2)	0.004 (2)	0.000 (2)	0.003 (2)
C3	0.021 (3)	0.022 (2)	0.018 (3)	0.001 (2)	−0.002 (2)	0.000 (2)
C4	0.019 (3)	0.022 (3)	0.025 (3)	0.000 (2)	0.004 (2)	0.003 (2)
C5	0.014 (2)	0.027 (3)	0.022 (3)	0.003 (2)	0.004 (2)	0.005 (2)
C6	0.024 (3)	0.014 (2)	0.028 (3)	0.004 (2)	0.001 (2)	0.001 (2)
C7	0.035 (3)	0.026 (3)	0.039 (3)	−0.007 (2)	−0.002 (3)	−0.003 (3)
C8	0.017 (2)	0.027 (3)	0.021 (3)	−0.001 (2)	−0.002 (2)	0.003 (2)
C9	0.028 (3)	0.024 (3)	0.034 (3)	−0.001 (2)	0.001 (2)	−0.001 (2)
C10	0.023 (3)	0.036 (3)	0.036 (3)	0.007 (2)	0.011 (2)	0.002 (3)
C11	0.038 (4)	0.054 (4)	0.021 (3)	0.008 (3)	−0.005 (3)	0.014 (3)
C12	0.013 (3)	0.061 (4)	0.035 (3)	−0.001 (3)	−0.002 (2)	0.015 (3)
C13	0.027 (3)	0.035 (3)	0.026 (3)	−0.003 (2)	−0.003 (2)	−0.001 (2)
C14	0.038 (4)	0.043 (3)	0.023 (3)	−0.017 (3)	−0.006 (3)	−0.003 (3)
C15	0.039 (4)	0.037 (3)	0.030 (3)	−0.014 (3)	−0.001 (3)	−0.005 (3)
C16	0.050 (4)	0.033 (3)	0.048 (4)	−0.006 (3)	0.010 (4)	−0.008 (3)
C17	0.038 (4)	0.059 (4)	0.026 (3)	−0.006 (3)	−0.002 (3)	−0.003 (3)
C18	0.070 (5)	0.041 (4)	0.024 (3)	−0.018 (3)	−0.017 (3)	−0.011 (3)
C19	0.082 (6)	0.031 (3)	0.036 (4)	−0.008 (4)	0.009 (4)	−0.001 (3)
C20	0.046 (4)	0.037 (3)	0.034 (4)	0.000 (3)	−0.007 (3)	0.005 (3)
C21	0.022 (3)	0.064 (4)	0.020 (3)	−0.007 (3)	0.011 (2)	0.000 (3)
C23	0.025 (3)	0.048 (3)	0.019 (3)	0.003 (3)	−0.008 (2)	0.004 (3)
C24	0.034 (3)	0.047 (4)	0.033 (3)	0.000 (3)	0.000 (3)	−0.010 (3)
C25	0.023 (3)	0.059 (4)	0.021 (3)	−0.005 (3)	−0.011 (2)	0.001 (3)
C26	0.022 (3)	0.053 (4)	0.036 (4)	0.003 (3)	0.001 (3)	0.005 (3)
C27	0.029 (3)	0.043 (3)	0.027 (3)	−0.004 (3)	0.013 (3)	0.005 (3)
C35	0.030 (3)	0.030 (3)	0.045 (4)	0.004 (3)	−0.018 (3)	−0.020 (3)
C36	0.043 (4)	0.043 (4)	0.037 (4)	−0.001 (3)	0.001 (3)	−0.005 (3)
C37	0.025 (3)	0.027 (3)	0.033 (3)	−0.002 (2)	−0.004 (3)	0.011 (2)
C38	0.038 (4)	0.035 (3)	0.030 (3)	0.006 (3)	−0.007 (3)	−0.008 (3)
C39	0.037 (4)	0.044 (4)	0.056 (5)	−0.015 (3)	−0.010 (4)	0.000 (3)
C40	0.043 (4)	0.047 (4)	0.045 (4)	−0.018 (3)	0.004 (3)	−0.001 (3)
C41	0.086 (6)	0.035 (3)	0.040 (4)	−0.005 (4)	0.023 (4)	0.004 (3)
C42	0.065 (5)	0.030 (3)	0.022 (3)	−0.006 (3)	−0.008 (3)	−0.002 (2)
C43	0.025 (3)	0.035 (3)	0.016 (3)	−0.005 (2)	−0.011 (2)	0.004 (2)
C44	0.035 (3)	0.031 (3)	0.025 (3)	0.000 (2)	−0.002 (3)	−0.002 (2)
C45	0.049 (4)	0.033 (3)	0.047 (4)	0.002 (3)	−0.012 (3)	−0.014 (3)
C46	0.033 (3)	0.045 (4)	0.033 (3)	−0.007 (3)	0.000 (3)	−0.006 (3)
C47	0.031 (4)	0.055 (4)	0.056 (5)	−0.005 (3)	−0.009 (3)	−0.012 (3)
C48	0.027 (3)	0.051 (4)	0.026 (3)	−0.012 (3)	0.008 (3)	−0.003 (3)
C49	0.039 (4)	0.027 (3)	0.042 (4)	0.011 (3)	−0.016 (3)	−0.003 (3)
C50	0.041 (4)	0.039 (3)	0.039 (4)	0.002 (3)	0.001 (3)	−0.007 (3)
C51	0.027 (3)	0.046 (4)	0.056 (4)	0.002 (3)	0.013 (3)	−0.012 (3)
C52	0.083 (6)	0.045 (4)	0.045 (4)	0.032 (4)	−0.006 (4)	0.006 (3)
C53	0.028 (3)	0.031 (3)	0.049 (4)	0.004 (2)	−0.015 (3)	0.014 (3)
C54	0.075 (6)	0.028 (3)	0.063 (5)	−0.013 (3)	0.011 (5)	0.020 (3)
C55	0.060 (4)	0.026 (3)	0.021 (3)	0.011 (3)	0.004 (3)	−0.003 (2)
C56	0.095 (7)	0.046 (4)	0.037 (4)	0.006 (4)	−0.036 (4)	−0.016 (3)

### Geometric parameters (Å, º)

**Table:**

P1—O2	1.450 (4)	C25—H25	0.9500
P1—O4	1.552 (4)	C26—C27	1.393 (9)
P1—O3	1.559 (4)	C26—H26	0.9500
P1—O1	1.593 (4)	C27—H27	0.9500
P2—O6	1.448 (4)	C35—H35A	0.9900
P2—O5	1.556 (4)	C35—H35B	0.9900
P2—O7	1.564 (4)	C36—H36A	0.9800
P2—O8	1.571 (4)	C36—H36B	0.9800
Si1—O11	1.659 (4)	C36—H36C	0.9800
Si1—C37	1.876 (6)	C37—C38	1.375 (9)
Si1—C43	1.878 (6)	C37—C42	1.407 (9)
Si1—C49	1.900 (7)	C38—C39	1.391 (10)
O1—C1	1.445 (6)	C38—H38	0.9500
O3—C14	1.425 (7)	C39—C40	1.397 (11)
O4—C7	1.482 (7)	C39—H39	0.9500
O5—C2	1.458 (6)	C40—C41	1.413 (11)
O7—C28A	1.461 (10)	C40—H40	0.9500
O7—C28B	1.53 (2)	C41—C42	1.357 (11)
O8—C21	1.467 (7)	C41—H41	0.9500
O9—C35	1.384 (7)	C42—H42	0.9500
O9—C3	1.410 (6)	C43—C48	1.399 (8)
O10—C36	1.427 (8)	C43—C44	1.433 (9)
O10—C35	1.430 (7)	C44—C45	1.399 (9)
O11—C4	1.434 (6)	C44—H44	0.9500
O12—C53	1.384 (7)	C45—C46	1.367 (10)
O12—C5	1.415 (6)	C45—H45	0.9500
O13—C53	1.414 (7)	C46—C47	1.385 (10)
O13—C54	1.448 (9)	C46—H46	0.9500
O14—C55	1.404 (7)	C47—C48	1.383 (9)
O14—C6	1.414 (6)	C47—H47	0.9500
O15—C55	1.401 (8)	C48—H48	0.9500
O15—C56	1.405 (9)	C49—C51	1.501 (10)
C1—C6	1.498 (7)	C49—C52	1.544 (10)
C1—C2	1.524 (7)	C49—C50	1.556 (9)
C1—H1	1.0000	C50—H50A	0.9800
C2—C3	1.554 (7)	C50—H50B	0.9800
C2—H2	1.0000	C50—H50C	0.9800
C3—C4	1.533 (7)	C51—H51A	0.9800
C3—H3	1.0000	C51—H51B	0.9800
C4—C5	1.531 (7)	C51—H51C	0.9800
C4—H4	1.0000	C52—H52A	0.9800
C5—C6	1.545 (7)	C52—H52B	0.9800
C5—H5	1.0000	C52—H52C	0.9800
C6—H6	1.0000	C53—H53A	0.9900
C7—C8	1.486 (8)	C53—H53B	0.9900
C7—H7A	0.9900	C54—H54A	0.9800
C7—H7B	0.9900	C54—H54B	0.9800
C8—C13	1.375 (8)	C54—H54C	0.9800
C8—C9	1.378 (8)	C55—H55A	0.9900
C9—C10	1.421 (8)	C55—H55B	0.9900
C9—H9	0.9500	C56—H56A	0.9800
C10—C11	1.399 (9)	C56—H56B	0.9800
C10—H10	0.9500	C56—H56C	0.9800
C11—C12	1.359 (10)	C28A—C29A	1.506 (13)
C11—H11	0.9500	C28A—H28A	0.9899
C12—C13	1.419 (9)	C28A—H28B	0.9900
C12—H12	0.9500	C30A—C31A	1.381 (9)
C13—H13	0.9500	C30A—C29A	1.393 (9)
C14—C15	1.474 (8)	C30A—H30A	0.9500
C14—H14A	0.9900	C31A—C32A	1.379 (9)
C14—H14B	0.9900	C31A—H31A	0.9500
C15—C16	1.384 (10)	C32A—C33A	1.388 (9)
C15—C20	1.414 (10)	C32A—H32A	0.9500
C16—C17	1.398 (9)	C33A—C34A	1.392 (9)
C16—H16	0.9500	C33A—H33A	0.9500
C17—C18	1.374 (11)	C34A—C29A	1.3771
C17—H17	0.9500	C34A—H34A	0.9500
C18—C19	1.336 (11)	C28B—C29B	1.47 (3)
C18—H18	0.9500	C28B—H28C	0.99 (3)
C19—C20	1.395 (9)	C28B—H28D	0.99 (3)
C19—H19	0.9500	C30B—C29B	1.391 (10)
C20—H20	0.9500	C30B—C31B	1.401 (10)
C21—C22	1.460 (9)	C30B—H30B	0.9500
C21—H21A	0.9900	C31B—C32B	1.393 (10)
C21—H21B	0.9900	C31B—H31B	0.9500
C22—C23	1.388 (9)	C32B—C33B	1.387 (10)
C22—C27	1.415 (9)	C32B—H32B	0.9500
C23—C24	1.395 (9)	C33B—C34B	1.392 (10)
C23—H23	0.9500	C33B—H33B	0.9500
C24—C25	1.380 (9)	C34B—C29B	1.389 (10)
C24—H24	0.9500	C34B—H34B	0.9500
C25—C26	1.374 (10)		
			
O2—P1—O4	117.3 (2)	O9—C35—H35A	108.9
O2—P1—O3	117.8 (3)	O10—C35—H35A	108.9
O4—P1—O3	99.4 (2)	O9—C35—H35B	108.9
O2—P1—O1	113.8 (2)	O10—C35—H35B	108.9
O4—P1—O1	102.3 (2)	H35A—C35—H35B	107.7
O3—P1—O1	103.9 (2)	O10—C36—H36A	109.5
O6—P2—O5	118.6 (2)	O10—C36—H36B	109.5
O6—P2—O7	114.6 (2)	H36A—C36—H36B	109.5
O5—P2—O7	102.5 (2)	O10—C36—H36C	109.5
O6—P2—O8	111.4 (2)	H36A—C36—H36C	109.5
O5—P2—O8	102.1 (2)	H36B—C36—H36C	109.5
O7—P2—O8	106.2 (2)	C38—C37—C42	117.3 (6)
O11—Si1—C37	109.3 (2)	C38—C37—Si1	120.5 (5)
O11—Si1—C43	110.5 (2)	C42—C37—Si1	121.8 (5)
C37—Si1—C43	109.0 (3)	C37—C38—C39	121.4 (6)
O11—Si1—C49	102.3 (3)	C37—C38—H38	119.3
C37—Si1—C49	111.0 (3)	C39—C38—H38	119.3
C43—Si1—C49	114.5 (3)	C38—C39—C40	120.3 (7)
C1—O1—P1	119.1 (3)	C38—C39—H39	119.9
C14—O3—P1	122.7 (4)	C40—C39—H39	119.9
C7—O4—P1	123.5 (4)	C39—C40—C41	118.8 (6)
C2—O5—P2	121.4 (3)	C39—C40—H40	120.6
C28A—O7—P2	120.8 (4)	C41—C40—H40	120.6
C28B—O7—P2	115.3 (9)	C42—C41—C40	119.0 (7)
C21—O8—P2	122.5 (4)	C42—C41—H41	120.5
C35—O9—C3	115.7 (5)	C40—C41—H41	120.5
C36—O10—C35	115.0 (5)	C41—C42—C37	123.1 (7)
C4—O11—Si1	125.3 (3)	C41—C42—H42	118.5
C53—O12—C5	118.5 (5)	C37—C42—H42	118.5
C53—O13—C54	109.8 (5)	C48—C43—C44	116.6 (5)
C55—O14—C6	114.8 (5)	C48—C43—Si1	124.4 (5)
C55—O15—C56	113.0 (6)	C44—C43—Si1	118.8 (4)
O1—C1—C6	108.9 (4)	C45—C44—C43	119.7 (6)
O1—C1—C2	107.9 (4)	C45—C44—H44	120.2
C6—C1—C2	109.8 (4)	C43—C44—H44	120.2
O1—C1—H1	110.1	C46—C45—C44	121.2 (7)
C6—C1—H1	110.1	C46—C45—H45	119.4
C2—C1—H1	110.1	C44—C45—H45	119.4
O5—C2—C1	108.0 (4)	C45—C46—C47	120.4 (6)
O5—C2—C3	107.6 (4)	C45—C46—H46	119.8
C1—C2—C3	109.4 (4)	C47—C46—H46	119.8
O5—C2—H2	110.6	C48—C47—C46	119.3 (6)
C1—C2—H2	110.6	C48—C47—H47	120.4
C3—C2—H2	110.6	C46—C47—H47	120.4
O9—C3—C4	110.6 (4)	C47—C48—C43	122.8 (6)
O9—C3—C2	109.5 (4)	C47—C48—H48	118.6
C4—C3—C2	107.4 (4)	C43—C48—H48	118.6
O9—C3—H3	109.8	C51—C49—C52	108.9 (6)
C4—C3—H3	109.8	C51—C49—C50	109.3 (6)
C2—C3—H3	109.8	C52—C49—C50	107.9 (6)
O11—C4—C5	110.1 (4)	C51—C49—Si1	113.2 (4)
O11—C4—C3	108.5 (4)	C52—C49—Si1	109.2 (5)
C5—C4—C3	107.5 (4)	C50—C49—Si1	108.2 (5)
O11—C4—H4	110.2	C49—C50—H50A	109.5
C5—C4—H4	110.2	C49—C50—H50B	109.5
C3—C4—H4	110.2	H50A—C50—H50B	109.5
O12—C5—C4	107.8 (4)	C49—C50—H50C	109.5
O12—C5—C6	110.7 (4)	H50A—C50—H50C	109.5
C4—C5—C6	109.7 (4)	H50B—C50—H50C	109.5
O12—C5—H5	109.6	C49—C51—H51A	109.5
C4—C5—H5	109.6	C49—C51—H51B	109.5
C6—C5—H5	109.6	H51A—C51—H51B	109.5
O14—C6—C1	111.2 (4)	C49—C51—H51C	109.5
O14—C6—C5	109.2 (4)	H51A—C51—H51C	109.5
C1—C6—C5	108.1 (4)	H51B—C51—H51C	109.5
O14—C6—H6	109.5	C49—C52—H52A	109.5
C1—C6—H6	109.5	C49—C52—H52B	109.5
C5—C6—H6	109.5	H52A—C52—H52B	109.5
O4—C7—C8	109.5 (4)	C49—C52—H52C	109.5
O4—C7—H7A	109.8	H52A—C52—H52C	109.5
C8—C7—H7A	109.8	H52B—C52—H52C	109.5
O4—C7—H7B	109.8	O12—C53—O13	114.0 (5)
C8—C7—H7B	109.8	O12—C53—H53A	108.8
H7A—C7—H7B	108.2	O13—C53—H53A	108.8
C13—C8—C9	120.3 (5)	O12—C53—H53B	108.8
C13—C8—C7	117.5 (5)	O13—C53—H53B	108.8
C9—C8—C7	122.2 (5)	H53A—C53—H53B	107.6
C8—C9—C10	119.4 (6)	O13—C54—H54A	109.5
C8—C9—H9	120.3	O13—C54—H54B	109.5
C10—C9—H9	120.3	H54A—C54—H54B	109.5
C11—C10—C9	119.5 (6)	O13—C54—H54C	109.5
C11—C10—H10	120.3	H54A—C54—H54C	109.5
C9—C10—H10	120.3	H54B—C54—H54C	109.5
C12—C11—C10	120.7 (6)	O15—C55—O14	111.8 (5)
C12—C11—H11	119.6	O15—C55—H55A	109.3
C10—C11—H11	119.6	O14—C55—H55A	109.3
C11—C12—C13	119.4 (6)	O15—C55—H55B	109.3
C11—C12—H12	120.3	O14—C55—H55B	109.3
C13—C12—H12	120.3	H55A—C55—H55B	107.9
C8—C13—C12	120.6 (6)	O15—C56—H56A	109.5
C8—C13—H13	119.7	O15—C56—H56B	109.5
C12—C13—H13	119.7	H56A—C56—H56B	109.5
O3—C14—C15	110.0 (5)	O15—C56—H56C	109.5
O3—C14—H14A	109.7	H56A—C56—H56C	109.5
C15—C14—H14A	109.7	H56B—C56—H56C	109.5
O3—C14—H14B	109.7	O7—C28A—C29A	105.4 (7)
C15—C14—H14B	109.7	O7—C28A—H28A	113.3
H14A—C14—H14B	108.2	O7—C28A—H28B	87.3
C16—C15—C20	118.5 (6)	O7—C28A—H28C	109 (10)
C16—C15—C14	122.1 (6)	C31A—C30A—C29A	117.4 (10)
C20—C15—C14	119.3 (6)	C31A—C30A—H30A	121.3
C15—C16—C17	120.1 (7)	C29A—C30A—H30A	121.3
C15—C16—H16	120.0	C32A—C31A—C30A	123.6 (11)
C17—C16—H16	120.0	C32A—C31A—H31A	118.2
C18—C17—C16	120.2 (7)	C30A—C31A—H31A	118.2
C18—C17—H17	119.9	C31A—C32A—C33A	119.1 (11)
C16—C17—H17	119.9	C31A—C32A—H32A	120.5
C19—C18—C17	120.6 (6)	C33A—C32A—H32A	120.5
C19—C18—H18	119.7	C32A—C33A—C34A	117.4 (10)
C17—C18—H18	119.7	C32A—C33A—H33A	121.3
C18—C19—C20	121.3 (7)	C34A—C33A—H33A	121.3
C18—C19—H19	119.4	C29A—C34A—C33A	123.4 (7)
C20—C19—H19	119.4	C29A—C34A—H34A	118.3
C19—C20—C15	119.3 (7)	C33A—C34A—H34A	118.3
C19—C20—H20	120.4	C34A—C29A—C30A	119.0 (7)
C15—C20—H20	120.4	C34A—C29A—C28A	120.8 (5)
C22—C21—O8	110.6 (5)	C30A—C29A—C28A	119.9 (9)
C22—C21—H21A	109.5	C29B—C28B—O7	110.0 (19)
O8—C21—H21A	109.5	C29B—C28B—H28C	106 (10)
C22—C21—H21B	109.5	O7—C28B—H28C	106 (10)
O8—C21—H21B	109.5	C29B—C28B—H28D	115 (10)
H21A—C21—H21B	108.1	O7—C28B—H28D	90 (10)
C23—C22—C27	119.2 (6)	H28C—C28B—H28D	129
C23—C22—C21	121.0 (6)	C29B—C30B—C31B	124 (2)
C27—C22—C21	119.7 (6)	C29B—C30B—H30B	117.8
C22—C23—C24	121.1 (6)	C31B—C30B—H30B	117.8
C22—C23—H23	119.4	C32B—C31B—C30B	109.9 (19)
C24—C23—H23	119.4	C32B—C31B—H31B	125.0
C25—C24—C23	118.8 (6)	C30B—C31B—H31B	125.0
C25—C24—H24	120.6	C33B—C32B—C31B	130 (2)
C23—C24—H24	120.6	C33B—C32B—H32B	114.9
C26—C25—C24	121.4 (6)	C31B—C32B—H32B	114.9
C26—C25—H25	119.3	C32B—C33B—C34B	115 (2)
C24—C25—H25	119.3	C32B—C33B—H33B	122.4
C25—C26—C27	120.4 (6)	C34B—C33B—H33B	122.4
C25—C26—H26	119.8	C29B—C34B—C33B	119 (2)
C27—C26—H26	119.8	C29B—C34B—H34B	120.3
C26—C27—C22	119.0 (6)	C33B—C34B—H34B	120.3
C26—C27—H27	120.5	C34B—C29B—C30B	120 (2)
C22—C27—H27	120.5	C34B—C29B—C28B	128.5 (19)
O9—C35—O10	113.5 (5)	C30B—C29B—C28B	111.3 (18)
			
O2—P1—O1—C1	−41.0 (4)	C16—C15—C20—C19	−1.3 (10)
O4—P1—O1—C1	−168.6 (4)	C14—C15—C20—C19	−178.3 (6)
O3—P1—O1—C1	88.3 (4)	P2—O8—C21—C22	−122.4 (5)
O2—P1—O3—C14	−15.3 (6)	O8—C21—C22—C23	88.3 (7)
O4—P1—O3—C14	112.5 (5)	O8—C21—C22—C27	−87.3 (7)
O1—P1—O3—C14	−142.2 (5)	C27—C22—C23—C24	−0.8 (9)
O2—P1—O4—C7	−44.4 (5)	C21—C22—C23—C24	−176.4 (6)
O3—P1—O4—C7	−172.5 (4)	C22—C23—C24—C25	0.3 (10)
O1—P1—O4—C7	80.9 (4)	C23—C24—C25—C26	−1.0 (10)
O6—P2—O5—C2	−25.0 (5)	C24—C25—C26—C27	2.3 (10)
O7—P2—O5—C2	102.3 (4)	C25—C26—C27—C22	−2.7 (9)
O8—P2—O5—C2	−147.8 (4)	C23—C22—C27—C26	2.0 (9)
O6—P2—O7—C28A	−22.2 (6)	C21—C22—C27—C26	177.7 (5)
O5—P2—O7—C28A	−152.0 (6)	C3—O9—C35—O10	−93.8 (6)
O8—P2—O7—C28A	101.2 (6)	C36—O10—C35—O9	83.7 (7)
O6—P2—O7—C28B	24.7 (11)	O11—Si1—C37—C38	−28.4 (6)
O5—P2—O7—C28B	−105.1 (11)	C43—Si1—C37—C38	−149.3 (5)
O8—P2—O7—C28B	148.1 (11)	C49—Si1—C37—C38	83.7 (5)
O6—P2—O8—C21	−165.0 (4)	O11—Si1—C37—C42	159.5 (5)
O5—P2—O8—C21	−37.5 (5)	C43—Si1—C37—C42	38.7 (6)
O7—P2—O8—C21	69.6 (5)	C49—Si1—C37—C42	−88.4 (6)
C37—Si1—O11—C4	−60.1 (4)	C42—C37—C38—C39	−1.9 (10)
C43—Si1—O11—C4	59.8 (5)	Si1—C37—C38—C39	−174.3 (5)
C49—Si1—O11—C4	−177.9 (4)	C37—C38—C39—C40	0.4 (11)
P1—O1—C1—C6	−114.4 (4)	C38—C39—C40—C41	1.7 (11)
P1—O1—C1—C2	126.5 (4)	C39—C40—C41—C42	−2.0 (11)
P2—O5—C2—C1	114.5 (4)	C40—C41—C42—C37	0.4 (11)
P2—O5—C2—C3	−127.5 (4)	C38—C37—C42—C41	1.5 (10)
O1—C1—C2—O5	−62.9 (5)	Si1—C37—C42—C41	173.8 (6)
C6—C1—C2—O5	178.5 (4)	O11—Si1—C43—C48	88.0 (5)
O1—C1—C2—C3	−179.8 (4)	C37—Si1—C43—C48	−151.9 (5)
C6—C1—C2—C3	61.6 (6)	C49—Si1—C43—C48	−26.8 (6)
C35—O9—C3—C4	139.1 (5)	O11—Si1—C43—C44	−86.4 (5)
C35—O9—C3—C2	−102.8 (5)	C37—Si1—C43—C44	33.7 (5)
O5—C2—C3—O9	61.0 (5)	C49—Si1—C43—C44	158.8 (5)
C1—C2—C3—O9	178.1 (4)	C48—C43—C44—C45	2.8 (9)
O5—C2—C3—C4	−178.9 (4)	Si1—C43—C44—C45	177.6 (5)
C1—C2—C3—C4	−61.8 (5)	C43—C44—C45—C46	−2.0 (10)
Si1—O11—C4—C5	−92.7 (5)	C44—C45—C46—C47	0.1 (11)
Si1—O11—C4—C3	149.9 (4)	C45—C46—C47—C48	0.9 (11)
O9—C3—C4—O11	−59.3 (5)	C46—C47—C48—C43	0.0 (11)
C2—C3—C4—O11	−178.7 (4)	C44—C43—C48—C47	−1.8 (10)
O9—C3—C4—C5	−178.4 (4)	Si1—C43—C48—C47	−176.3 (5)
C2—C3—C4—C5	62.2 (5)	O11—Si1—C49—C51	−47.2 (5)
C53—O12—C5—C4	122.3 (5)	C37—Si1—C49—C51	−163.7 (5)
C53—O12—C5—C6	−117.8 (5)	C43—Si1—C49—C51	72.3 (5)
O11—C4—C5—O12	−61.0 (5)	O11—Si1—C49—C52	−168.7 (5)
C3—C4—C5—O12	57.1 (5)	C37—Si1—C49—C52	74.8 (6)
O11—C4—C5—C6	178.5 (4)	C43—Si1—C49—C52	−49.2 (6)
C3—C4—C5—C6	−63.4 (5)	O11—Si1—C49—C50	74.1 (5)
C55—O14—C6—C1	−109.5 (5)	C37—Si1—C49—C50	−42.4 (5)
C55—O14—C6—C5	131.4 (5)	C43—Si1—C49—C50	−166.4 (4)
O1—C1—C6—O14	61.7 (6)	C5—O12—C53—O13	85.6 (6)
C2—C1—C6—O14	179.6 (4)	C54—O13—C53—O12	65.8 (8)
O1—C1—C6—C5	−178.5 (4)	C56—O15—C55—O14	−72.3 (7)
C2—C1—C6—C5	−60.5 (6)	C6—O14—C55—O15	−73.3 (7)
O12—C5—C6—O14	64.5 (6)	C28B—O7—C28A—C29A	60.1 (15)
C4—C5—C6—O14	−176.7 (4)	P2—O7—C28A—C29A	154.4 (5)
O12—C5—C6—C1	−56.6 (6)	C29A—C30A—C31A—C32A	5 (2)
C4—C5—C6—C1	62.2 (5)	C30A—C31A—C32A—C33A	−2 (2)
P1—O4—C7—C8	171.3 (4)	C31A—C32A—C33A—C34A	0.6 (19)
O4—C7—C8—C13	177.5 (5)	C32A—C33A—C34A—C29A	−2.1 (16)
O4—C7—C8—C9	−3.9 (8)	C33A—C34A—C29A—C30A	4.8 (9)
C13—C8—C9—C10	−1.2 (9)	C33A—C34A—C29A—C28A	178.4 (15)
C7—C8—C9—C10	−179.8 (5)	C31A—C30A—C29A—C34A	−5.9 (15)
C8—C9—C10—C11	−0.1 (9)	C31A—C30A—C29A—C28A	−179.6 (11)
C9—C10—C11—C12	−0.2 (9)	O7—C28A—C29A—C34A	119.2 (4)
C10—C11—C12—C13	1.7 (9)	O7—C28A—C29A—C30A	−67.3 (13)
C9—C8—C13—C12	2.7 (9)	C28A—O7—C28B—C29B	−68.7 (17)
C7—C8—C13—C12	−178.6 (5)	P2—O7—C28B—C29B	−177.4 (13)
C11—C12—C13—C8	−2.9 (9)	C29B—C30B—C31B—C32B	4 (4)
P1—O3—C14—C15	−170.9 (4)	C30B—C31B—C32B—C33B	−4 (4)
O3—C14—C15—C16	−99.6 (7)	C31B—C32B—C33B—C34B	7 (4)
O3—C14—C15—C20	77.4 (7)	C32B—C33B—C34B—C29B	−9 (3)
C20—C15—C16—C17	−1.2 (10)	C33B—C34B—C29B—C30B	9 (4)
C14—C15—C16—C17	175.8 (6)	C33B—C34B—C29B—C28B	−172 (3)
C15—C16—C17—C18	2.4 (10)	C31B—C30B—C29B—C34B	−7 (4)
C16—C17—C18—C19	−1.1 (11)	C31B—C30B—C29B—C28B	174 (2)
C17—C18—C19—C20	−1.4 (12)	O7—C28B—C29B—C34B	67 (3)
C18—C19—C20—C15	2.6 (11)	O7—C28B—C29B—C30B	−114 (2)

### Hydrogen-bond geometry (Å, º)

*Cg*1 is the centroid of the C8–C13 ring.

**Table:**

*D*—H···*A*	*D*—H	H···*A*	*D*···*A*	*D*—H···*A*
C19—H19···O13^i^	0.95	2.50	3.342 (9)	148
C32*A*—H32*A*···O10^ii^	0.95	2.30	3.243 (11)	173
C14—H14*B*···*Cg*1^iii^	0.99	2.86	3.832 (7)	168

Symmetry codes: (i) *x*+1/2, −*y*+1/2, −*z*+1; (ii) −*x*+1, −*y*+1, *z*; (iii) *x*−1/2, −*y*+1/2, −*z*+2.
